# Accumulating Mutations in Series of Haplotypes at the *KIT* and *MITF* Loci Are Major Determinants of White Markings in Franches-Montagnes Horses

**DOI:** 10.1371/journal.pone.0075071

**Published:** 2013-09-30

**Authors:** Bianca Haase, Heidi Signer-Hasler, Matthew M. Binns, Gabriela Obexer-Ruff, Regula Hauswirth, Rebecca R. Bellone, Dominik Burger, Stefan Rieder, Claire M. Wade, Tosso Leeb

**Affiliations:** 1 Faculty of Veterinary Science, University of Sydney, Sydney, New South Wales, Australia; 2 Institute of Genetics, Vetsuisse Faculty, University of Bern, Bern, Switzerland; 3 Department of Agriculture, Forestry, Food Science and Management, Bern University of Applied Science, Zollikofen, Switzerland; 4 Equine Analysis, Midway, Kentucky, United States of America; 5 Department of Biology, University of Tampa, Tampa, Florida, United States of America; 6 Swiss Institute of Equine Medicine, ALP-Haras and University of Bern, Avenches, Switzerland; 7 Agroscope ALP-Haras Research Station, Swiss National Stud Farm, Avenches, Switzerland; 8 DermFocus, University of Bern, Bern, Switzerland; University of Colorado, School of Medicine, United States of America

## Abstract

Coat color and pattern variations in domestic animals are frequently inherited as simple monogenic traits, but a number are known to have a complex genetic basis. While the analysis of complex trait data remains a challenge in all species, we can use the reduced haplotypic diversity in domestic animal populations to gain insight into the genomic interactions underlying complex phenotypes. White face and leg markings are examples of complex traits in horses where little is known of the underlying genetics. In this study, Franches-Montagnes (FM) horses were scored for the occurrence of white facial and leg markings using a standardized scoring system. A genome-wide association study (GWAS) was performed for several white patterning traits in 1,077 FM horses. Seven quantitative trait loci (QTL) affecting the white marking score with p-values *p*≤10^−4^ were identified. Three loci, *MC1R* and the known white spotting genes, *KIT* and *MITF*, were identified as the major loci underlying the extent of white patterning in this breed. Together, the seven loci explain 54% of the genetic variance in total white marking score, while *MITF* and *KIT* alone account for 26%. Although *MITF* and *KIT* are the major loci controlling white patterning, their influence varies according to the basic coat color of the horse and the specific body location of the white patterning. Fine mapping across the *MITF* and *KIT* loci was used to characterize haplotypes present. Phylogenetic relationships among haplotypes were calculated to assess their selective and evolutionary influences on the extent of white patterning. This novel approach shows that *KIT* and *MITF* act in an additive manner and that accumulating mutations at these loci progressively increase the extent of white markings.

## Introduction

A major challenge in the post-genome era is the handling and analysis of complex traits. At this time the understanding of inter-locus allelic interactions is limited and the interpretations of computational results can be complicated. Various genome-wide association studies in humans, comprising of thousands of samples, have worked with complex phenotypes aiming to identify causal variants that explain a high proportion of variance in observed phenotypes. The significantly associated variants are often observed to explain only a small fraction of the estimated total additive genetic variance [1]–[3].

The long history of artificial selection in domestic animal populations has created unique model populations that facilitate the study of complex and quantitative traits [4]. Coat colors in animals have been popular model traits in genetics as the phenotypes are relatively easy to assess, and of broad public interest. The outcomes of coat color studies have already provided insight into the functions of melanistic genes, signaling pathways and epistatic interactions [5]–[9].

White markings and other de-pigmentation patterns are caused by either a lack of melanocytes due to an incomplete formation and migration of melanocyte precursor cells during embryonic development (leucism), or by the inability of melanocytes to produce pigment (albinism) [10], [11]. There appears to have been a steady rise in the occurrence of de-pigmentation and color variation phenotypes in domestic animals, presumably as a result of domestication [12]–[15]. It is believed that white markings and de-pigmentation patterns were favored as a means of both identifying owned individuals and distinguishing them from their wild relatives. Despite the practical usefulness of markings and their aesthetic desirability, pelage de-pigmentation is frequently associated with undesirable side effects, including neurological defects and eye disorders [7], [16]–[19].

In many domestic species, it is appreciated that de-pigmentation patterns are under the control of several loci, including *TYR*, *MITF*, *KIT*, *EDNRB*, *SILV, PAX3* [17], [19], [20]–[28], and the genes above are typically studied as functional candidate genes by researchers studying white patterning phenotypes. While it is known that these genes and others affect pigmentation in many mammalian species, it is interesting to consider whether as yet unidentified loci might also play a role in the distribution of white markings in the equine and other species. Various independent studies have shown that the extent of white markings in horses is highly heritable (h^2^>0.5) and it has been previously demonstrated that the horse’s basic coat color has a significant impact on the expression of white [29]–[31]. Based on segregation analyses, Woolf [30], [32]–[35] concluded that white markings exhibit a complex mode of inheritance and that environmental factors contribute to the occurrence of white markings in horses. Differences in de-pigmentation patterns between monozygotic twins provide evidence that factors independent of nuclear genetics exert an influence on the amount of white [36]. Similarly, epigenetic modifications have been shown to influence the phenotypic expression of white markings in mice [37].

In the Franches-Montagnes (FM) horse population the average extent of white markings has steadily increased during the past thirty years, despite a breed standard that calls for a horse with little or no white markings. Segregation analyses indicated that white markings have a dominant-recessive mode of inheritance at a bi-allelic locus with an additional polygenic effect [31]. A strong positive correlation between the melanocortin receptor locus (*MC1R*) and white markings has been established, with pheomelanistic chestnut horses having a trend towards more extensive white patterning than other base coat colors [19], [33], [34].

This study investigates the molecular genetics underlying white facial and leg markings in FM horses. We hypothesize that an accumulation of mutations rather than a single new mutation event is responsible for extended white facial and leg markings, and therefore, we have applied a novel approach to explore the phylogenetic relationships among haplotypes in the vicinity of candidate genes of major effect.

## Results

### Genome-wide Association Study for the Amount of White

**Figure 1:**
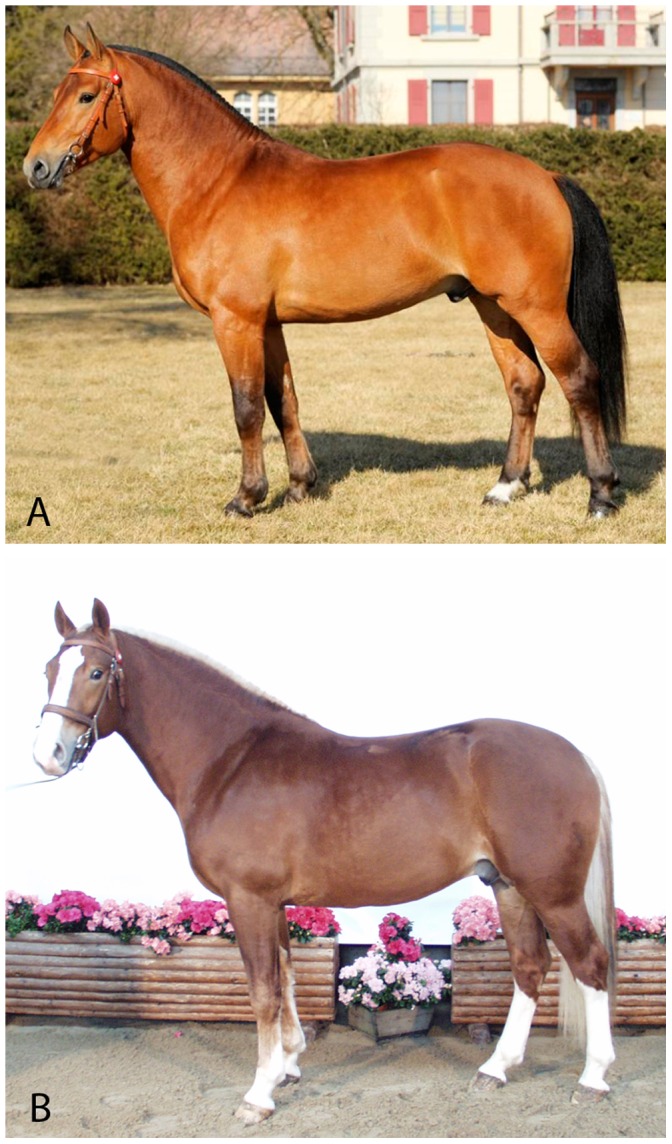
Phenotypic variation in the expression of white markings. Example of phenotypes. Horse (A) has a total score of white markings of 1 (head = 0; foreleg = 0; hind leg = 1); horse (B) has a total score of white markings of 19 (head = 9; foreleg = 2; hind leg = 8).

**Figure 2:**
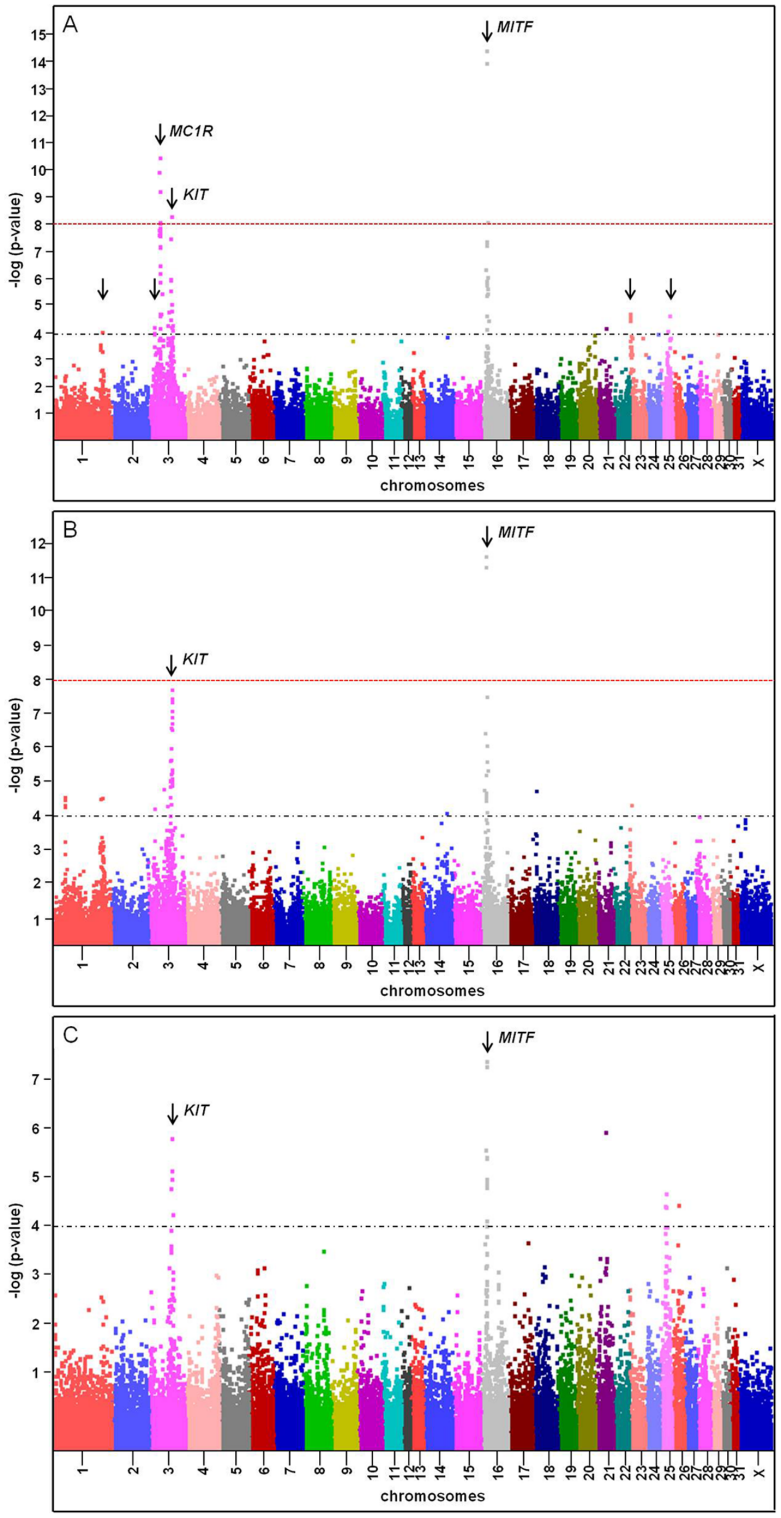
GWAS identifies two major loci associated with total white marking scores. A Manhattan plot showing the negative log of the probability of association (p-value) between individual marker and total white marking score. (A) Analysis included horses of all colors, (B) bay horses only, (C) chestnut horses only. Markers are represented in different colors according to their chromosome. Significance level of p≤1×10^−8^ is indicated with a dashed red line; a dashed black line represents association with p≤10^−4^.

A set of 1,077 horses was selected from the Swiss FM horse population [38]. The amounts of white on the head, forelegs, and hind legs were estimated using a previously described scoring system and were then combined to give a score for either total white or for different body locations (head, forelegs, hind legs; Figure 1) [31]. Genotypes were determined using the Illumina 50 k equine genotyping array. A genome-wide association analysis using EMMAX [39] identified seven loci significantly (*p*≤10^−4^) associated with the total amount of white (Figure 2, Table S1). The most significantly associated SNP is located on ECA 16 within intron 1B of the *MITF* gene (*p*≤10^−15^). The second most associated locus is adjacent to *MC1R* and a third highly significant locus, also located on ECA3, is in close proximity to the *KIT* gene. Other loci with significant associations for total white markings scores are located on ECA1, 3, 23, and 25, respectively. Association analyses were conducted within and across basic coat color groups (chestnut or bay; Figure S1 (1–12)). We examined loci affecting quantitative phenotypes based on combined scores from different body locations (total, head, foreleg and hind leg; Figure S1 (1–12)). While analyses for all body locations and basic coat colors confirmed *MITF* and *KIT* as the two major loci, an associated locus on ECA25 reached significance (*p* = 3⋅10^−6^) for the amount of white on the forelegs in chestnut horses only (Figure S1–11; Table S1). The observed associations on ECA1 and ECA23 were specific to bay horses and showed highest associations for white on the hind legs (*p* = 6⋅10^−6^) and forelegs (*p* = 6⋅10^−7^), respectively (Figure S1–7, S1–8; Table S1). SNPs at all seven associated loci in this analysis were also found to explain a substantial proportion (54%) of the genetic variance in total white marking score.

When all horses were analyzed together, the entire chromosomes ECA3 and ECA16 explained 38% and 17%, respectively (data not shown), of the observed genetic variance in total white marking score while the SNPs in close proximity (within 2 Mb) of *KIT* (ECA3) and *MITF* (ECA16) explained 13% and 14% of the genetic variance in total white markings score, respectively (Table 1). Thus, while *MITF* is responsible for the majority of the signal derived from ECA16, other genes on ECA3 (including *MC1R*) play a role in the expression of total white markings on horses. White markings on the heads of FM horses appear to be influenced by *MITF* and *MC1R* rather than by *KIT* (Table 1). *MITF* accounts for 23% of the genetic variance in white head markings in all horses, while *KIT* accounts only for 10% of the genetic variance in this trait. In chestnut horses (*MC1R* fixed), *MITF* accounts for 41% of the variance in white head markings, and *KIT*, 22% of the genetic variance. Leg markings (mean of fore- and hind leg scores) in the FM breed are driven predominantly by ECA3 with *KIT* and *MC1R* explaining 10% of the variation, respectively. *MITF* explains on average 5% of variation in leg markings across all horses, but considerably more in bay horses.

**Table 1:** Genetic variance (%) explained by the three major candidate loci.

		CHR3						CHR16			
		*MC1R*	SE	*KIT*	SE	remainingSNPs	SE	*MITF*	SE	remainingSNPs	SE
**all horses**	head	36.4	11.3	10.2	4.2	6.2	2.9	22.6	<0.0	1.5	<0.0
n = 1077	fore-leg	8.5	<0.0	6.2	<0.0	10.9	<0.0	6.7	4.3	6.0	5.0
	hind leg	10.9	7.6	13.0	5.7	0.6	4.2	3.5	<0.0	1.8	<0.0
	total score	17.5	10.4	12.1	5.0	8.3	3.7	13.6	5.4	2.3	2.5
**bay horses**	head	2.6	<0.0	12.8	<0.0	19.9	<0.0	22.8	9.2	3.7	4.0
n = 749	fore-leg	1.5	<0.0	6.4	<0.0	27.4	<0.0	19.2	9.7	3.0	6.8
	hind leg	2.1	<0.0	11.2	<0.0	4.4	<0.0	5.2	4	2.4	5.2
	total score	1.6	<0.0	13.3	<0.0	19.8	<0.0	20.4	<0.0	1.2	<0.0
**chestnut horses**	head	0.8	<0.0	21.6	<0.0	3.3	<0.0	41.0	15.5	4.1	5.7
n = 328	fore-leg	1.0	<0.0	10.0	<0.0	1.6	<0.0	3.3	4	6.0	7.9
	hind leg	0.8	<0.0	19.0	<0.0	2.2	<0.0	4.9	4.6	6.2	7.7
	total score	0.4	<0.0	18.4	<0.0	1.5	<0.0	11.8	6.2	7.2	5.6

The contributions of *MC1R*, *KIT* and *MITF* were calculated separately for each locus across a 2 Mb interval.

### Fine Mapping and Re-sequencing

**Figure 3:**
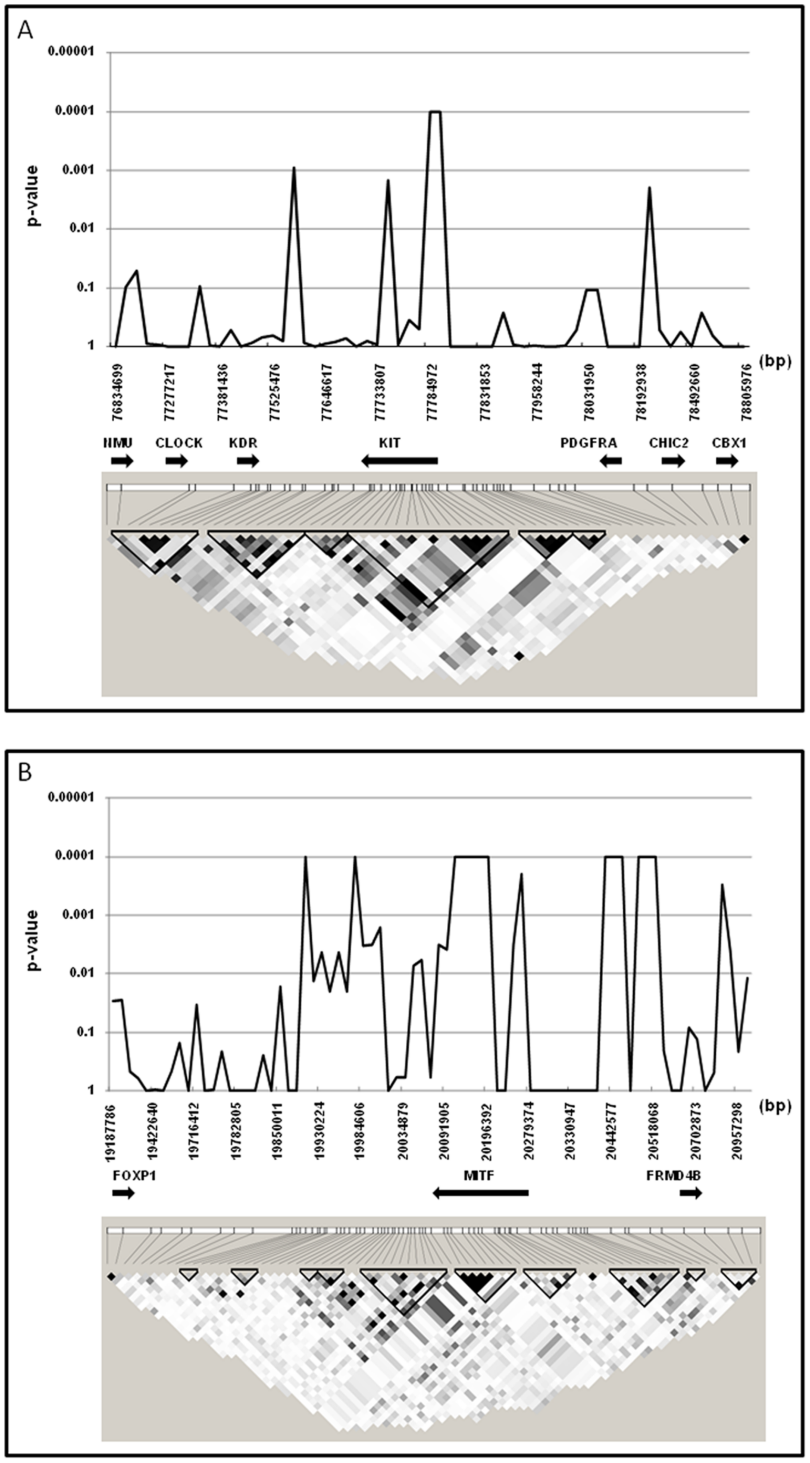
Fine-scale quantitative association mapping and linkage disequilibrium across *MITF* and *KIT*. Fine–scale quantitative association between total white markings score and linkage disequilibrium (LD) in r^2^ between SNPs across 2 Mb regions in (A) the *KIT* and (B) the *MITF* region including all horses. The darker shading represents higher LD, black diamond’s represents an r^2^ of 1.

A set of 383 FM horses were used to investigate the fine-scale associations of the *MITF* and *KIT* loci with total white markings score. A total of 96 single nucleotide polymorphisms spread over a 2 Mb interval for each of the *MITF* and *KIT* loci were genotyped using a custom designed Illumina GoldenGate assay (Table S2). Results from the individual quantitative association analyses of the *KIT* and the *MITF* loci are shown in figure 3 (and Figure S2). The most highly associated interval at the *KIT* locus was located within intron 1 between 77,784,972 bp and 77,785,750 bp. While bay horses showed diffuse signals of association across both loci, chestnut horses showed strong association at the *KIT* locus only. Although several SNPs showed high association with total white markings at the *MITF* locus, the longest block of adjacent SNPs with high association was located within the *MITF* gene and the region of association was even more distinct if the analysis included bay horses only, consistent with *MITF* influence on leg markings in bay horses as described above. The 99 kb region of refined association at the *MITF* locus was represented by seven SNPs in this analysis and spanned the genomic region between 20,111,359 bp and 20,210,729 bp, which includes the interval between intron 1 H and intron 4 of the *MITF* gene. Re-sequencing of the entire 99 kb interval of strongest consistent association in two bay horses identified a total of 151 variants between the two individuals (Table S3). These novel variants were genotyped in the same set of 383 fine mapping horses. Of these newly identified variants 59 were excluded from further analyses as they failed the quality and frequency checks. The remaining 92 SNP markers were combined with genotypes from the fine mapping analysis, resulting in a total of 177 markers available for analysis over the 2 Mb *MITF* interval.

### Phylogenetic Relationships of Haplotypes and the Affect on White Markings Score

Haplotype block structures across both fine mapped intervals (*KIT* and *MITF*) were defined in Haploview [40] and each individual’s haplotype phases determined using PLINK [41]. A quantitative association analysis using PLINK [41] identified a haplotype with a permutation-derived p-value of *p*≤10^−4^ in every haplotype block (data not shown). When the results were fitted with a linear regression model, the interval between 20,026,912 bp and 20,223,433 bp on ECA 16 was identified as the major haplotype block for the *MITF* locus, and the interval on ECA3 from 77,733,807 bp to 77,934,490 bp as the major haplotype block for the *KIT* locus. While the major haplotype block on ECA16 was located within the *MITF* gene, the haplotype block on ECA3 spanned 95% of the *KIT* gene including 5′-flanking sequence. Six different haplotypes termed M1–M6 were observed across *MITF*, and 7 different haplotypes, termed K1–K7 were observed for *KIT* (Table 2; Table S4 *)*.

**Table 2:** Results of the quantitative association analysis within the major haplotype block.

	haplotype	frequency (%)	beta coefficient	*p*-value	EMP2
MITF	M1	20.9	−4.620	0.0000	0.0001
	M2	14.1	0.079	0.9440	1.0000
	M3	7.6	−0.127	0.9290	1.0000
	M4	14.1	1.100	0.2540	0.9913
	M5	7.5	4.600	0.0004	0.0067
	M6	8.6	6.440	0.0000	0.0001
KIT	K1	11.0	−3.690	0.0002	0.0029
	K2	6.1	0.164	0.9010	1.0000
	K3	28.6	−1.260	0.0784	0.5114
	K4	11.7	2.260	0.0220	0.1717
	K5	23.9	1.590	0.0374	0.2802
	K6	5.9	0.273	0.8320	1.0000
	K7	5.3	1.940	0.1790	0.8133

Results of the haplotype-based quantitative association analysis within the major haplotype block of *MITF* (M1–M6) and *KIT* (K1–K7). The analysis includes all horses used for fine mapping. Haplotype frequency, beta coefficient, p-value and empirical p-value are indicated for every investigated haplotype.

**Figure 4:**
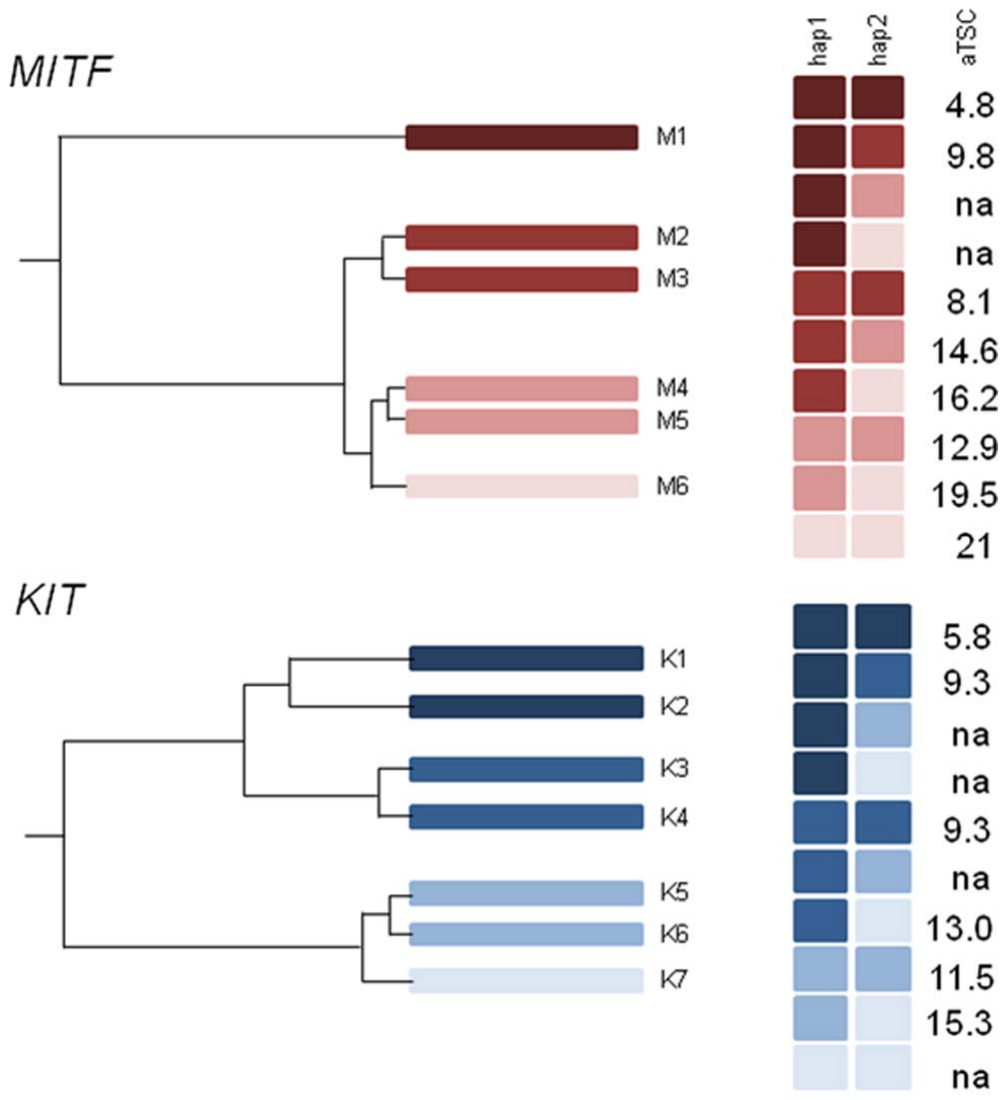
Phylogenetic relationship between haplotypes at *MITF* and *KIT*. Phylogenetic relationships between haplotypes at *MITF* (M1–M6) and *KIT* (K1–K7). Haplotype combinations (hap1 = haplotype 1, hap2 = haplotype 2) and individuals average total white markings score (aTSC = average Total Score) are shown on the right.

**Figure 5:**
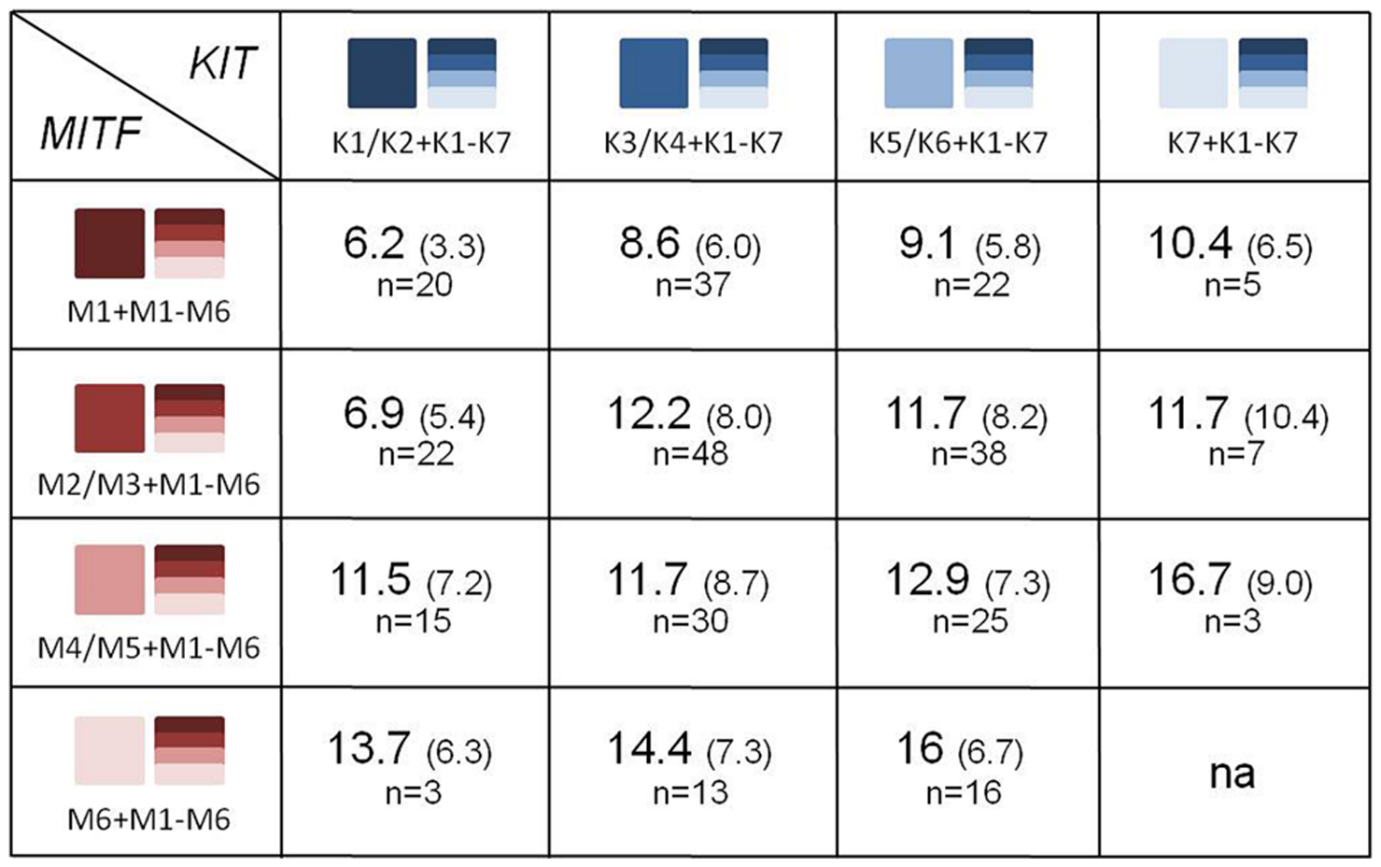
Average total white markings score for *MITF-KIT* haplotype combinations. Average total white markings score and standard deviation for *MITF-KIT* haplotype combinations. Adjacent squares represent haplotypes (red = *MITF*; blue = *KIT*); color shades represent haplotypes of the phylogenetic relationship trees (*MITF*: M1–M6; *KIT*: K1–K7).

The quantitative association analysis using PLINK [41] showed that *MITF* haplotypes M1, M5 and M6 were significantly associated with total white markings score (Table 2). While M1 was negatively correlated with total white markings score, M5 and M6 showed positive correlations with total white markings. At the major *KIT* haplotype block, only haplotype K1 showed significant association (*p* = 0.0029) and was negatively correlated with total white marking score. These results suggest protective effects of haplotypes M1 and K1 on the occurrence of total white markings. Phylogenetic relationships of haplotypes and the mean total white marking scores for haplotype combinations within each locus were calculated (Figure 4) and results showed a clear additive haplotype effect and highlighted the protective effects of haplotypes M1 (*MITF*), K1 and K2 (*KIT*). While the *MITF* haplotype combinations accounted for mean total white marking scores ranging from 4.8 to 21, haplotype combinations at the *KIT* explained a smaller range of mean total white markings scores between 5.8 and 15.3. These observations suggest that *MITF* haplotypes have larger effects on total white markings scores. To investigate the effects of *MITF-KIT* haplotype combinations on the total white marking scores we included only horses that had fully phased haplotypes exhibiting the major associated haplotypes for both the *MITF* and *KIT* haplotype blocks. Such complete information was available for 110 horses. This analysis enabled us to demonstrate additive haplotype effects within and across loci (Figure 5).

**Figure 6:**
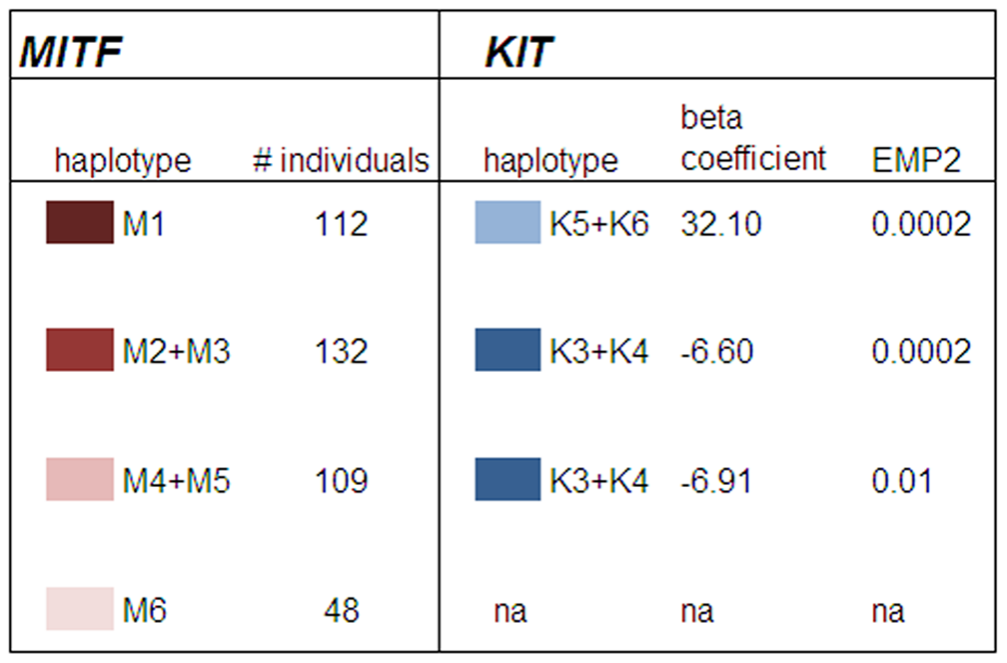
Results of the logistic regression model analysis. Results of the logistic regression model analysis for the relationship between *MITF* haplotypes, SNPs across the 2 Mb *KIT* region *and KIT* haplotypes.

When *MITF* haplotypes M1–M6 were included as fixed effects, the haplotype-based logistic regression model analyses were able to estimate the effects of *KIT* (haplotypes K1–K7) on the total white markings score (Figure 6). A single haplotype at the *KIT* locus was shown to be significantly associated with total white markings scores in horses that had total white markings scores adjusted for the influences of *MITF* haplotypes M1–M5. Haplotypes K5 and K6 (*KIT*) were found to exert strong positive influences on total white markings scores in horses that are homozygous for the M1 haplotype (beta coefficient = 32.1, permutation–derived p-value = 2⋅10^−4^). No association was able to be calculated for *MITF* M6, due to the low frequency of this haplotype in the population assessed.

## Discussion

In this study the extent of white markings was quantified for 1,077 Franches-Montagnes horses and all horses were analyzed on the Illumina 50 k equine genotyping array. A genome-wide association study identified seven QTL for total white marking scores. All seven loci together explain 54% of the genetic variation of total white marking scores; with *KIT* and *MITF* as the two major loci explaining 12% and 14%, respectively. Both genes have been investigated intensively in conjunction with de-pigmentation patterns. It has been shown in many studies that they are crucial for melanocyte development and pigment synthesis. Several independent studies have demonstrated that mutations in *MITF* and *KIT* cause a wide range of de-pigmentation phenotypes with varying phenotypic expressions [17], [19], [26], [28], [42], [43].

The GWA study identified four novel loci associated with total white marking scores (ECA1∶155,795,652 bp; ECA3∶19,281,146 bp; ECA23∶2,603,893 bp and ECA25∶29,621,832 bp). Interestingly, none of the additional identified QTL contains genes previously reported to be involved in de-pigmentation. While two genes *TRPM1* and *OCA2* known to cause de-pigmentation are located on ECA1 [44]–[46], these genes are located 45 Mb and 50 Mb upstream of the best associated SNPs in our analysis, thus it is unlikely that these genes are responsible for the QTL identified. The other QTL contain various genes involved in the regulation of cell differentiation and gene regulation, but none of the genes has previously been implicated to coat color phenotypes in any other species in the literature.

The proportion of genetic variance explained by chromosomes and candidate genes indicates that *KIT* predominantly modifies the amount of white markings in chestnut horses, while *MITF* predominantly modifies the amount of white in bay horses (Table 1). These results are confirmed by the fine mapping analyses. The chestnut phenotype is caused by a recessive *MC1R* loss-of function mutation which results in exclusive production of pheomelanistic pigments [47], [48]. This loss-of-function mutation is likely to inhibit melanogenic enzymes due to a down-regulation of intracellular signaling pathways, including *MITF* expression [49]. *MITF* expression is required early during embryonic development to allow neural crest cells to enter the melanocyte pathway as it promotes the transition of precursor cells [50]. Melanocyte development is aborted and cannot be rescued if *MITF* expression is lacking during this early stage. The dysfunctional melanocortin-1-receptor which inhibits the expression of *MITF* is likely to reduce the number of melanoblasts. A generally reduced number of melanocytes would make chestnut horses more susceptible to the effects of *KIT* mutations and would thereby explain the increased extent of white markings. Furthermore, it would explain why *KIT* appears to play a more important role in chestnut horses. The analysis also indicates that, regardless of the basic coat color, head markings are predominantly modified by *MITF*. This finding is supported by results from Rieder et al. [31] who showed that the genetic correlations between head markings and legs (forelegs and hind legs, respectively) is smaller than between forelegs and hind legs. It has been described that head and body melanocytes originate from different parts of the neural crest [51]. While melanoblasts derived from the trunk neural crest migrate dorsolaterally towards the ventral midline, cardiac neural crest cells migrate in a rostral orientation, producing structures of the head, including melanocytes. A likely scenario would be that the differentiation, migration and survival of these cell lines are differentially regulated.

As the extent of white markings has been shown to increase over the breeding history of the FM breed we hypothesized that multiple accumulating mutations were responsible for this increase. To verify our hypothesis we developed a novel approach that allowed us to calculate haplotype-specific effects on white marking scores based on the evolutionary history of haplotypes. A multiple regression analysis identified major haplotype blocks for *KIT* and *MITF*, respectively. One may argue that because information of only one haplotype block per locus was used for the calculation we disregarded information from other blocks. However, as the linear regression analysis indicated that none of the other haplotype blocks contributed significantly to the amount of white, the effect is likely to be marginal. Furthermore, the different haplotype combinations explain a large range of the average total amount of white markings; both results together give evidence that those haplotype blocks have the major effects on the investigated phenotype.

To calculate the effect of *MITF* and *KIT* haplotype combinations, phylogenetic information was used to group related haplotypes and analyze them together to achieve sufficient cohort sizes. The result of the grouping was to allow us to demonstrate a clear additive haplotype effect. It is possible that the grouping may have caused a loss in fine resolution but we expect this effect to be negligible. Even with the large number of horses in this analysis, individuals homozygous for haplotypes at *MITF* and *KIT* were rare. As a consequence, individuals with at least one of the investigated haplotypes were grouped together to calculate haplotype-specific effects meaning that heterozygous horses were often used more than once. Even with grouping, the average total white marking score was unable to be calculated for some haplotype combinations that did not exist in our dataset. Nevertheless, the results clearly demonstrate the presence of an additive haplotype effect, not only within one locus but also among the different *MITF-KIT* haplotype combinations.

In conclusion, our novel approach of applying phylogenetic relationships among haplotypes to study their effects on quantitative traits enabled us to more clearly understand some quite complex regulatory mechanisms. This approach allowed us to reveal substantial haplotypic diversity at the *KIT* and *MITF* loci. We are able to explain a large proportion of the genotypic variation in total white markings score in the FM breed and we show that *MITF* and *KIT* haplotypes act in an additive manner.

## Materials and Methods

### Ethic Statement

No experiments with animals was performed for this study except the collection of blood from the jugular vein by a licensed veterinarian or from hairs pulled form mane or tail by the horse owner or researcher. All animal work was conducted in accordance with the relevant local guidelines (Swiss law on animal protection and welfare - permit to the Swiss National Stud Farm no. 2227).

### Animals

A total of 1,077 Franches-Montagnes horses (749 bay and 328 chestnut) were selected and an EDTA stabilized blood sample was collected from every individual. DNA was extracted using standard methods. Every horse was genotyped using the Illumina 50 k equine genotyping array. The extent of white markings (head; foreleg; hind leg; total) was estimated for every horse based on a standardized scoring system as described before (Rieder et al. 2008) (Figure 1). As the presence or the extent of white markings of horses can be caused or influenced by sabino-1 (*KIT: c.2350-13T>A)* the absence of the sabino-1 variant was experimentally excluded in all 383 horses used for fine mapping.

### Genome-wide Association Study and Estimation of Genetic Variance Explained

The software PLINK [41] was used to convert.ped and.map files to EMMAX-readable files. Only SNPs with a minor allele frequency of ≥0.05 and a genotyping rate of ≥0.25 were included. Using the public available software efficient mixed-model association eXpedited (EMMAX) [39] the pairwise genetic relatedness matrix was calculated from genome wide high-density markers and a variance component model was subsequently used to estimate the restricted maximum likelihood parameters. Using the quantitative values for the amount of white, the calculation was performed for head, forelegs and hind legs separately as well as for the total white marking score. Calculated p-values of *p*≤10^−8^ were considered as genome-wide significant and p-values of *p*≤10^−4^ as suggestively significant. Subsequently, horses were separated based on basic coat color (bay and chestnut) and the analysis repeated. The program GCTA [52] was used to estimate the proportion of genetic variance explained by chromosomes and the seven QTLs, respectively. A genomic relationship matrix using SNP genotype information was built for each chromosome separately. A second genomic relationship matrix was constructed which included SNPs from all chromosomes excluding the chromosome investigated. The variance explained by chromosome was calculated with EM-REML and repeated until values did no longer change between iterations. To estimate the proportion of genetic variance explained by the seven QTLs a 2 Mb region covering the loci was used to build the first genomic relationship matrix followed by the second matrix including all array SNPs but excluding those of the first matrix. To build genomic relationship matrixes only SNPs with a minor allele frequency of at least 0.05 were included. All genomic positions given in this manuscript correspond to the EquCab 2 assembly.

### Fine Mapping and Re-sequencing

A set of 383 horses, consisting of 221 bay and 162 chestnut horses was selected to fine map the candidate loci on ECA3 and ECA16. Horses were selected to represent the full range of total white marking scores. All horses were genotyped for *MC1R*:c.901C>T using PCR-RFLP with *Taq*l according to Marklund et al. [47] to confirm basic coat color. A custom Illumina GoldenGate genotyping assay was developed based on public SNP information for *MITF* and *KIT*, respectively. A set of 96 SNPs spanning a total interval of 2 megabases (Mb) was selected; SNPs were selected with an average space of 1SNP/10 kb across the candidate gene and 1SNP/50 kp for gene flanking regions. After quality and frequency check using the software GenomeStudio (Illumina, Inc., San Diego, CA) a total of 91 markers were available for *MITF* and 82 markers for *KIT*. Genotyping results were analyzed using the software PLINK [41] with total white marking scores as quantitative values. Only SNPs with a minor allele frequency of ≥0.05 were included in the analysis and 10,000 permutations were performed to obtain an empirical significance threshold. Pairwise linkage disequilibrium patterns for the fine mapped regions of *MITF* and *KIT* were generated in r^2^ using Haploview [40]. SNPs with a minor allele frequency of <0.05 were excluded for the calculation.

The entire 99 kb *MITF* region associated with the total white marking score was sequenced in two bay horses. Overlapping PCR products were generated and analyzed on an ABI3730 capillary sequencer. Both horses were homozygous wild-type at *MC1R* and showed an identical haplotype over the fine mapped *MITF* region. One horse had a total score of 3 (minimal white markings) while the other horse had a total score of 21 (extended white markings). For regions with repetitive elements or high GC content long-range PCR using SequalPrep (Invitrogen) was performed and products were sequenced using the 454 FLX sequencing technology (Roche). A total of 151 polymorphisms were identified (Table S3) and subsequently genotyped in all 383 horses, either using a custom Illumina GoldenGate genotyping assay (Table S2) or targeted Sanger re-sequencing. The Bead Studio Data Analysis Software v 3.2.33 (Illumina) was used for a cluster analysis of SNPs genotyped with the GoldenGate assay. Polymorphisms with insufficient clustering were excluded from subsequent analyses.

### Identification of Haplotype Blocks, Calculation of Phylogenetic Relationships and Interactions Between Haplotypes

SNPs from fine-scale mapping and re-sequencing with a genotyping frequency of ≥0.85 were combined and used to define haplotype blocks for *MITF* and *KIT* in Haploview [40] using the confidence interval by Gabriel et al. [53]. Individual haplotype phases for the in Haploview identified haplotype blocks were determined for horses previously used in fine mapping using PLINK [41]. A haplotype-based quantitative association analysis was performed for haplotypes of every haplotype block separately using PLINK performing 10,000 permutations. Only haplotypes with a minor frequency of ≥0.05 were included in the analysis. To estimate effects between haplotype blocks a linear regression analysis was applied for each locus including significantly associated haplotypes as fixed effect. The analysis was performed using the software PLINK and 10,000 permutations were performed to obtain an empirical significance threshold.

Phylogenetic relationships among haplotypes of the major *MITF* and *KIT* haplotype blocks were calculated with the phylogenetic analysing package PHYLIP version 3.6 [54], including haplotypes with a minor frequency of at least 0.05. Distance matrixes between haplotypes were calculated with phylip dnadist and phylogenetic relationships were estimated using the Fitch-Margoliash distance matrix method with molecular clock, with negative branch length not allowed. Closely related haplotypes were grouped and the average total white marking score was calculated for different haplotype combinations. To estimate the effect of different *MITF-KIT* haplotype combinations on the total white marking score, individuals carrying at least one of the investigated haplotypes were combined and the average amount of total white markings was calculated. A haplotype based general logistic regression model analysis using PLINK was applied to calculate haplotype effects of the *KIT* locus including *MITF* haplotypes as fixed effects. The result was corrected for multiple testing with 10,000 permutations.

## Supporting Information

Figure S1 **(1–12): Results of the genome-wide association study.** Manhattan plots showing the negative log of the probability of association (p-value) between individual marker and white marking scores. Markers are represented in different colors according to their chromosome. Significance level of p≤1×10^−8^ is indicated with a dashed red line; a dashed black line represents association with p≤10^−4^.
(TIF)

Figure S2 **Fine-scale quantitative association mapping and linkage disequilibrium across** ***MITF*** **and** ***KIT.*** Fine–scale quantitative association between total white markings score and linkage disequilibrium (LD) between SNPs across 2 Mb regions in (1) the *KIT* and (2) the *MITF* region including (A) bay horses only and (B) chestnut horses only.
(TIF)

Table S1 **NPs of the Illumina equine SNP50 genotyping array with significant association to white marking scores.**
(XLSX)

Table S2 **Polymorphisms used for fine scale mapping of** ***KIT*** **(ECA3) and** ***MITF*** **(ECA16).**
(XLSX)

Table S3 **Variants identified in the re-sequenced** ***MITF*** **interval.**
(XLSX)

Table S4 **Haplotypes and genomic position of SNPs defining the major** ***KIT*** **and** ***MITF*** **haplotype blocks.**
(XLSX)
