# Supplementary figures and images for: Accumulating Mutations in Series of Haplotypes at the KIT and MITF Loci Are Major Determinants of White Markings in Franches-Montagnes Horses
Source: PLoS One. 2013 Sep 30;8(9):e75071. doi: 10.1371/journal.pone.0075071 (PMC3787084; doi:10.1371/journal.pone.0075071)

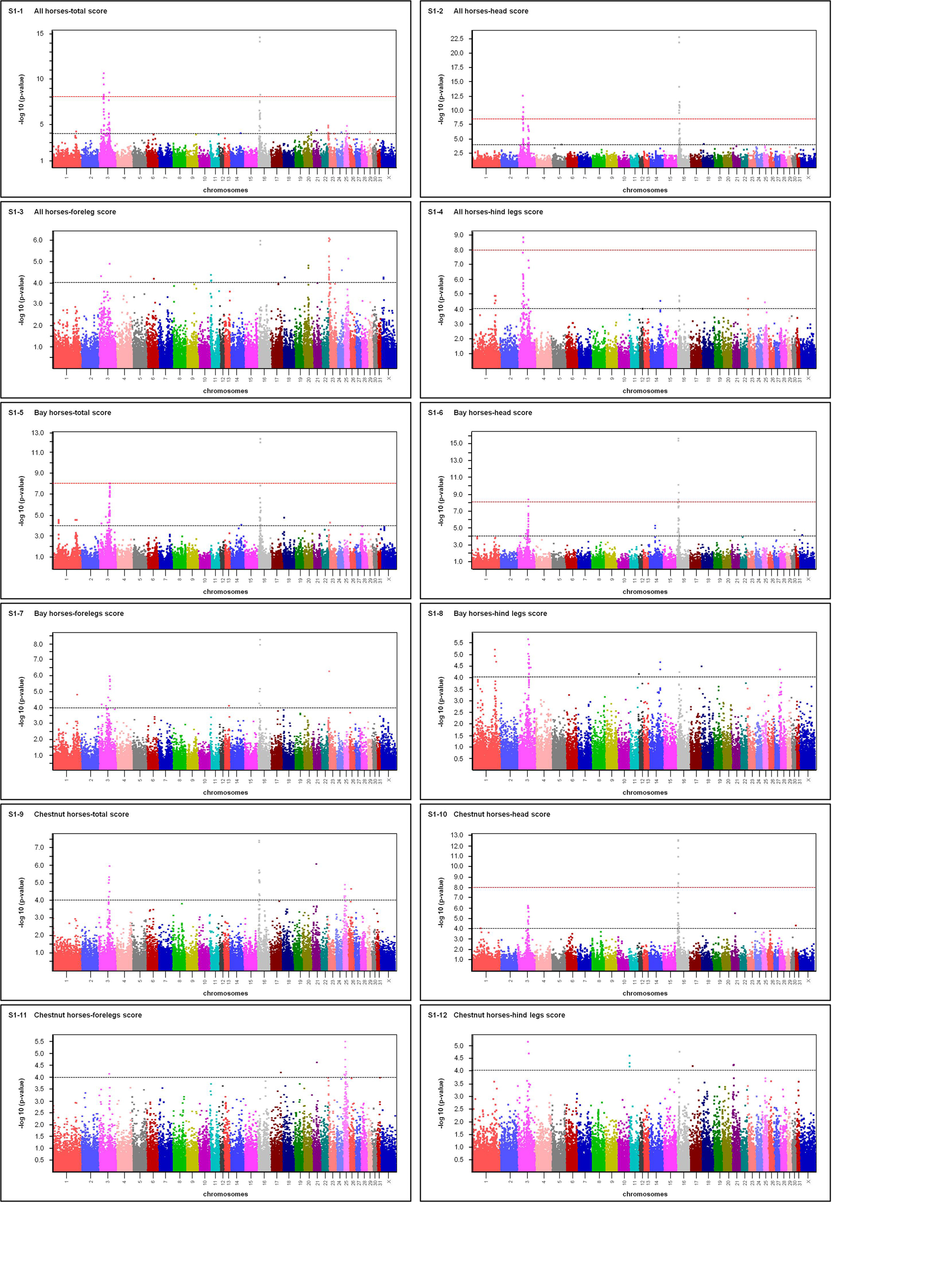

Supplement: Figure S1 — (1–12): Results of the genome-wide association study. Manhattan plots showing the negative log of the probability of association (p-value) between individual marker and white marking scores. Markers are represented in different colors according to their chromosome. Significance level of p≤1×10^−8^ is indicated with a dashed red line; a dashed black line represents association with p≤10−4. (TIF) [file pone.0075071.s001.tif]

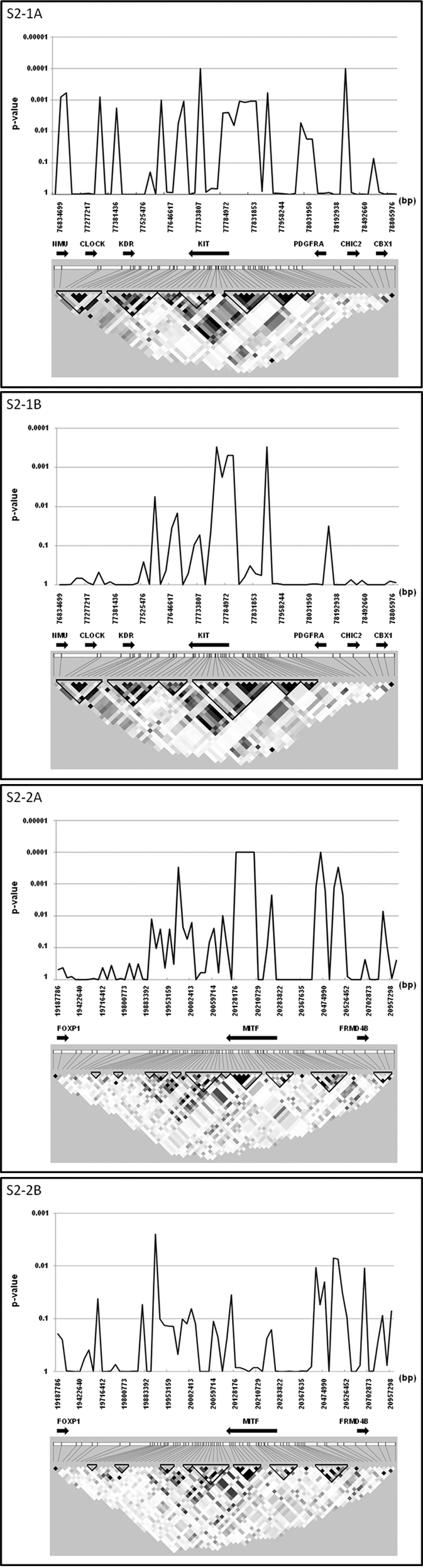

Supplement: Figure S2 — Fine-scale quantitative association mapping and linkage disequilibrium across MITF and KIT. Fine–scale quantitative association between total white markings score and linkage disequilibrium (LD) between SNPs across 2 Mb regions in (1) the KIT and (2) the MITF region including (A) bay horses only and (B) chestnut horses only. (TIF) [file pone.0075071.s002.tif]
